# Edible flora in pre-Columbian Caribbean coprolites: Expected and unexpected data

**DOI:** 10.1371/journal.pone.0292077

**Published:** 2023-10-11

**Authors:** Jelissa Reynoso-García, Tasha M. Santiago-Rodriguez, Yvonne Narganes-Storde, Raul J. Cano, Gary A. Toranzos

**Affiliations:** 1 Environmental Microbiology Laboratory, Biology Department, University of Puerto Rico, San Juan, Puerto Rico; 2 Diversigen, Inc., New Brighton, Minnesota, United States of America; 3 Center for Archaeological Research, University of Puerto Rico, San Juan, Puerto Rico; 4 Biological Sciences Department, California Polytechnic State University, San Luis Obispo, California, United States of America; New York State Museum, UNITED STATES

## Abstract

Coprolites, or mummified feces, are valuable sources of information on ancient cultures as they contain ancient DNA (aDNA). In this study, we analyzed ancient plant DNA isolated from coprolites belonging to two pre-Columbian cultures (Huecoid and Saladoid) from Vieques, Puerto Rico, using shotgun metagenomic sequencing to reconstruct diet and lifestyles. We also analyzed DNA sequences of putative phytopathogenic fungi, likely ingested during food consumption, to further support dietary habits. Our findings show that pre-Columbian Caribbean cultures had a diverse diet consisting of maize (*Zea mays*), sweet potato (*Ipomoea batatas*), chili peppers (*Capsicum annuum*), peanuts (*Arachis* spp.), papaya (*Carica papaya*), tomato (*Solanum lycopersicum*) and, very surprisingly cotton (*Gossypium barbadense*) and tobacco (*Nicotiana sylvestris*). Modelling of putative phytopathogenic fungi and plant interactions confirmed the potential consumption of these plants as well as edible fungi, particularly *Ustilago* spp., which suggest the consumption of maize and huitlacoche. These findings suggest that a variety of dietary, medicinal, and hallucinogenic plants likely played an important role in ancient human subsistence and societal customs. We compared our results with coprolites found in Mexico and the United States, as well as present-day faeces from Mexico, Peru, and the United States. The results suggest that the diet of pre-Columbian cultures resembled that of present-day hunter-gatherers, while agriculturalists exhibited a transitional state in dietary lifestyles between the pre-Columbian cultures and larger scale farmers and United States individuals. Our study highlights differences in dietary patterns related to human lifestyles and provides insight into the flora present in the pre-Columbian Caribbean area. Importantly, data from ancient fecal specimens demonstrate the importance of ancient DNA studies to better understand pre-Columbian populations.

## Introduction

The Huecoid and the Saladoid, two pre-Columbian indigenous cultures, migrated from different regions of the Americas in independent migratory waves to settle in the Caribbean Islands [1–3]. Since its discovery, it has been hypothesized that each culture was distinct based on major differences in the pottery, lapidary and faunal cultural assemblage [3], and that both co-habited the site of Sorcé, Vieques, Puerto Rico for more than 1,000 years [4]. A counter hypothesis was presented in which the Huecoid materials belonged to the Saladoid tradition [5]. While the Saladoid culture is believed to have migrated from the Orinoco River Valley of Venezuela [6], and inhabited the island of Vieques around the sixth century B.C. [7], the Huecoid culture is believed to have originated on the eastern slopes of the Andean mountains of present-day Bolivia and Peru [8], and to have arrived to Puerto Rico around the third century B.C. In addition to differences in pottery and faunal materials, as well as migratory patterns, a considerable amount of lapidary objects consisting of semiprecious stone ornaments, which include jadeite condors distinguished the Huecoid culture and supports the proposed Andean origin of this culture [9, 10]. In contrast, the Saladoid culture was characterized by polychromic (white and orange over red) painted pottery tradition [6, 11], distinct faunal assemblage and significant shell ornament industry.

Highly developed phytocultural practices that connected these pre-Columbian cultures in the Caribbean to South America resulted in complex social systems [12]. Early European chroniclers [13, 14], and more recently starch remains stored on plant-processing artifacts (as well as human dental calculus) have shown a complex food system in the Caribbean [15, 16]. During the early ceramic age, the ancient South American and Caribbean Amerindians harvested a variety of plants including maize (*Zea mays*), sweet potato (*Ipomoea batatas)*, common bean (*Phaseolus vulgaris*), manioc (*Manihot esculenta*), marunguey (*Zamia* spp.), cocoyam (*Xanthosoma* sp.), and peanut (*Arachis hypogaea*) [15–17]. Minor dietary components included achira (*Cannaceae*) and arrowroot *(Maranta arundinacea)* [18], while chili peppers (*Capsicum* spp.) were used as a condiment. Many of these plants continued being used during the Late Ceramic Age, although indigenous people included a larger repertoire of plants (including fruits) [17, 19] and the importance of *Cannaceae* and *Marantaceae* increased. While paleoethnobotanical data are corroborating the information given by the Spanish chroniclers, significant gaps in knowledge of the pre-Columbian diet, and regional and temporal differences in dietary habits remain. The chroniclers described mainly the culture, flora and fauna of Hispaniola, holding the notion that all the Caribbean islands were alike. Thus, the information about Puerto Rico and other islands of the Caribbean is limited. In addition, the Huecoid and Saladoid are early undescribed cultures since the chroniclers only described later cultures. Information on consumed plants contributing to the diet of pre-Columbian and present-day ethnic groups with contrasting geocultural regions and temporal scales is needed to better understand diet as an important part of present culture and identity.

Mummified feces, or coprolites, recovered from archaeological sites have provided a wealth of valuable information about pre-Columbian diets and the past environment in which theses cultures lived [20]. For instance, micro- (e.g., pollen), and macroscopic remains (e.g., bones, seeds, and fibers) recovered from coprolites have provided dietary and lifestyle information [21, 22]. Similarly, the presence of pollen from famine foods in coprolites may suggest that the individual may have lived in arid environments [20, 23]. In addition to micro- and macroscopic remains, DNA analysis has revealed the diet of extinct sloths [24, 25], dogs [26, 27], moas [28, 29], and mummies [30, 31]. Moreover, the reconstruction of ancient human diets has been possible in part by studies of the gut microbiome [32, 33], virome [34], parasitome [35], and mycobiome [36] found in coprolites. Notably, ancient microbial communities detected in coprolites can also reflect the evolution of human lifestyles through time [23, 36–38]. Other DNA sequences, such as those originating from plants, may provide a refined taxonomic classification of edible plants, which, in turn, may aid in the reconstruction of ancient dietary habits and lifestyles.

Our study presents data on plant DNA extracted from coprolites (mummified feces) recovered from Vieques, Puerto Rico, which are approximately 1500 years old. Our goal was to reconstruct the diet and surrounding flora of the pre-Columbian Huecoid and Saladoid cultures using this plant DNA data. To better understand how diet varied across different geocultural regions and historical periods, we also analyzed coprolites from other cultures, including the Loma San Gabriel culture in La Cueva de los Muertos Chiquitos (Rio Zape, Mexico), the Ancestral Puebloans in the Arid West Cave (Arizona, USA), and the Boomerang Shelter (Utah, USA). Additionally, we included data obtained from present-day fecal samples from various indigenous groups, such as the Matses (hunter-gatherers from the Peruvian Amazon), traditional Tunapuco (agriculturalists from the Peruvian Andean highlands), and Mazahua (farmers from Mexico), as well as urban-industrial individuals from the United States. To further support our analysis of dietary habits, we investigated DNA sequences from phytopathogenic fungi, which can be ingested along with the corresponding plant species. Overall, our study aimed to use plant and fungal DNA extracted from coprolites to shed light on the ancient dietary habits of pre-Columbian cultures of Puerto Rico, and to identify any differences in diet across different geocultural and historical periods.

## Materials and methods

### Archaeological samples and site

Coprolites from the Huecoid and Saladoid cultures from La Hueca archeological site in Sorcé, Vieques (18º 05’ 56” Latitude North and 65º 29’ 34” Longitude West), a semi-arid island located about 13 km southeast of the main island of Puerto Rico were used. Archaeologists Luis Chanlatte and Yvonne Narganes conducted the excavations on private land with the owner’s approval and followed all relevant regulations. In total, ten coprolites from the two pre-Columbian cultures were used: six of the coprolites corresponded to the Huecoid culture and four of the coprolites corresponded to the Saladoid culture. Detailed information about the samples is presented in S1 Table. The collection is deposited at the Center for Archaeological Research at the University of Puerto Rico, San Juan, Puerto Rico. Specimen registration numbers have been previously described [35]. Coprolite age was estimated using radiocarbon dating from associated archeological material (charcoal and shells) [8]. All samples were carbon-dated at Teledyne Isotopes (Westwood, NJ) and BETA Analytic, Inc. (Miami, FL) using a standard protocol. Radiocarbon dating estimates for the Huecoid coprolite samples ranged from 245 to 600 A.D., whereas the Saladoid coprolites ranged from 230 to 395 A.D. [34]. Coprolites have yielded well-preserved gut microbiome DNA [34, 35] as well as human and plant DNA.

### DNA extraction and contamination prevention

DNA was extracted from all coprolite samples as previously described [34]. Briefly, ten coprolites from the Huecoid (*n* = 6) and Saladoid (*n* = 4) cultures were processed in a class II biological safety cabinet exclusively dedicated to ancient DNA following strict procedures: protective clothes, disinfection of surfaces, sterilization of instruments, and ultraviolet radiation. The class II biosafety cabinet was cleaned with 70% ethanol and exposed to ultraviolet radiation for 30 minutes before and after use. To avoid modern exogenous contamination, DNA extraction was performed using only the inner core of each coprolite after the removal of the exterior portion using sterile and flamed scalpels. Total DNA was extracted using the PowerSoil DNA extraction kit (Mo Bio Laboratories, Carlsbad, CA, USA) according to the manufacturer’s instructions. The inner core of the coprolites was pulverized using a sterile mortar and pestle and hydrated overnight in sterile C1 solution at 4 °C. Because of low concentrations of DNA, samples were then pooled into one composite sample per culture using standard glycogen precipitation protocols (Thermo Scientific).

### Metagenomic library construction and shotgun sequencing

Whole-genome amplification from small quantities of DNA was performed using a REPLI-g Midi kit (Qiagen). Amplified DNA was purified using the PowerClean DNAClean-Up Kit (MO BIO Laboratories) and sample concentrations were calculated using the Qubit® dsDNA HS Assay Kit (Life Technologies). Library preparation was completed using the Nextera DNA Sample Preparation kit (Illumina) according to the manufacturer’s recommendations. Libraries concentrations were evaluated using the Qubit® dsDNA HS Assay Kit (Life Technologies) and the average library size was quantified using Experion (Bio-Rad). Libraries were then pooled in equimolar ratios and shotgun sequenced on the Illumina Miseq sequencing platform at MR DNA Research lab (Shallowater, TX) [34].

### Comparison with other coprolite and present-day fecal sequences

Publicly available sequence data were obtained from the NCBI’s Sequence Read Archive (SRA) using the SRA Toolkit (v2.10.4). Archaeological samples constitute sequencing data from 13 coprolites, including coprolites from the Loma San Gabriel culture in La Cueva de Los Muertos Chiquitos (n = 8, Rio Zape, Mexico; under BioProject ID: PRJEB31971, PRJEB33577, and PRJEB35362) [23, 39, 40]; from the Ancestral Puebloans from the Arid West Cave (n = 3, Arizona, USA; BioProject ID: PRJNA561510) [23]; and from the Ancestral Puebloans from the Boomerang Shelter (n = 2, Utah, USA; BioProject ID: PRJNA561510) [23] (Table 1).

**Table 1:** Description of the archaeological samples analyzed in this study.

Pre-Columbian culture	Archaeological site	Geographical regions	Coprolite C-14 data (range)	Reference
**Huecoid**	La Hueca Sorcé	Vieques, Puerto Rico	1500 BP	This study, [34, 35]
**Saladoid**	La Hueca, Sorcé	Vieques, Puerto Rico	1500 BP	This study, [34, 35]
**Loma San Gabriel**	La Cueva de los Muertos Chiquitos, Rio Zape	Durango, Mexico	1300 BP	[39, 40]
**Puebloans**	Arid West Cave	Arizona, United States	2500–1500 BP	[23]
**Puebloans**	Boomerang Shelter	Utah, United States	2000–1000 BP	[23]

Present-day samples comprised published sequence data from 86 extant stools, including feces from the Matses hunter-gatherers (n = 24, Peru, BioProject ID: PRJNA268964) [41]; the Tunapuco farmers (n = 12, BioProject ID: PRJNA268964); the Mazahua farmers (n = 22, Mexico, BioProject ID: PRJNA561510) [23]; and United States individuals from the Human Microbiome Project (n = 28, USA, BioProject ID: PRJNA48479) [42] (Table 2). All samples were computationally analyzed along with the data from our study [43].

**Table 2:** Description of the published present-day samples analyzed in this study.

Present-day culture	N	Subsistence strategy	Geographical region	Reference
**Matses**	24	Hunter-gatherers	Peru	[41]
**Tunapuco**	12	Agriculturalists	Peru	[41]
**Mazahua**	22	Agriculturalists	Mexico	[23]
**United States**	28	Urban-industrial	United States	[42]

### Bioinformatics and statistical analysis

#### Read processing and quality control

Raw paired-end reads were trimmed and filtered from adapters and low-quality reads (Phred score < 20) through trim-galore using default parameters as implemented in the metaWRAP Read_qc module (v1.2.4) [44]. Contaminating human DNA sequences were then removed from the metagenomic datasets through alignment of reads to the *Homo sapiens* reference genome (build Hg38) using the BMTagger approach implemented in the metaWRAP Read_qc module [44]. The human origin of the coprolites was evaluated through the detection of human-specific *Bacteroides* by PCR, as presented in previous studies from our laboratory [33]. Quality control improvement on sequencing reads was assessed using FastQC [45]. Pre-processed reads were considered for all downstream analyses.

#### Metagenomic profiling

Taxonomic assignment of high-quality sequencing reads was performed through Kaiju as implemented in command-line (v1.5.0) [46] using the following parameters: −a greedy −E 0.05 for e-value filtering. Kaiju classified reads using a subset of the NCBI BLAST non-redundant (nr) reference database (argument -nr_euk) comprising annotated protein-coding genes from bacteria, archaea, viruses, and fungi (accessed on 25 May 2020). Taxon IDs from plant sequences from the NCBI nr database were also included. It has been shown that a database comprising all domains of life is better suited for taxonomic profiling of microbial eukaryotes [47].

#### Functional ecological guilds

Ecological functions (trophic and guilds) of fungal genera were parsed using FUNGuild (v.1.2) (https://github.com/UMNFuN/FUNGuild) [48]. Fungal genera that classified within the plant pathogen functional guild were considered for further analysis.

#### Source tracking of microbial communities

The proportion of DNA reads from each potential source contributing to the Huecoid and Saladoid sink coprolite samples was estimated using Meta-SourceTracker (mSourceTracker) [49]. Publicly available shotgun libraries from human feces, coprolites, and human skin were downloaded from the Sequence Read Archive (SRA) using SRA Toolkit (v2.10.4) and the soil metagenomes were downloaded from MG-RAST using grabseqs [50]. These reference metagenomes were selected as potential sources and contaminants (i.e., soil and skin) for coprolite samples. The environmental source samples included: 58 non-industrial human feces, 28 industrial feces, 13 coprolites, 16 human skin, and 16 soil samples. All samples were processed using the metagenome classifier Kaiju and then combined using the mSourceTracker script kaiju_table_to_OTU_table.py. The resulting table for the Eukaryotic domain was converted to HDF5 biom format using the biom-format python package (v.2.1.10) and then used as an input for mSourceTracker.

#### Plant-pathogen interaction network

We used the rglobi (global biotic interactions) R package to extract all the interactions between the plants and phytopathogenic fungi (queried as “Fungi”) in the dataset using the get_interactions_by_taxa function. In addition, we retrieved plant disease data from the American Phytopathological Society website (https://www.apsnet.org/edcenter/resources/commonnames/Pages/default.aspx). We only retained potential phytopathogenic fungi that were identified through Kaiju in our dataset. Pathogen-host interaction network was constructed from a taxonomic table by generating a directional data frame of pathogen-host interactions as identified using rglobi and plant disease data. We then imported the dataset table into Cytoscape to build a directed network. For the resulting network, we calculated the degree of connectivity, and the eigenvector centrality using CytoNCA, to assess the importance of each node.

#### Statistical analysis and visualization

Sequencing data were primarily analyzed and visualized using the R statistical environment, version v4.1.3 (R Foundation for Statistical Computing). For beta diversity, dimensional reduction of Aitchison distances was visualized in a principal coordinates analysis (PCoA) using the phyloseq R package (v.1.38.0) [51]. Statistical differences in beta diversity were tested through Permutational Multivariate Analysis of Variance (PERMANOVA) using the adonis function in the phyloseq R package. A hierarchical clustering dendrogram based on Bray-Curtis dissimilarity distances was constructed on plant abundance per sample using the Ward’s clustering algorithm in the vegan R package (v. 2.5.7) [52]. Maps and piedonut plots were generated using the R packages sf (v.1.0.7), webr (v.0.1.6), and ggplot2 (v. 3.3.5).

## Results

### General patterns of plant DNA in coprolites from the Huecoid and Saladoid

We studied ten coprolites recovered from an archaeological site in Vieques, Puerto Rico in an attempt to reconstruct dietary habits of the pre-Columbian Huecoid and Saladoid cultures. We analyzed DNA sequences from plants and their potential phytopathogenic fungi using shotgun metagenomic sequencing, which may contain fewer biases in ancient microbiome reconstruction compared to amplicon-based sequencing [53]. Following bioinformatic processing, plant sequence reads were classified into one phylum, one class, five orders, five families, eight genera, and nine species (Table 3). Six of the taxa have not been previously detected in paleoethnobotanical studies and thus are putative taxonomic assignments. In addition, the putative plant sequences identified could be a closely related plant taxon.

**Table 3:** Description of the identified taxa from DNA sequencing of the Huecoid and Saladoid coprolites.

Order	Family	Genus	Species	Common name	Possible Origin^a^	Uses
**Poales**	Poaceae	*Zea*	*Zea mays*	Maize	Mesoamerica	Foodstuff
**Brassicales**	Caricaceae	*Carica* *	*Carica papaya*	Papaya	Tropical America	Foodstuff
**Fabales**	Fabaceae	*Arachis* *	*Arachis hypogaea*	Domesticated peanut	Brazilian–Paraguayan Center	Foodstuff
**Fabales**	Fabaceae	*Arachis* *	*Arachis duranensis*	Wild peanut	Brazilian–Paraguayan Center	Foodstuff
**Solanales**	Solanaceae	*Ipomoea*	*Ipomoea batatas*	Sweet potato	Central America	Foodstuff
**Solanales**	Solanaceae	*Capsicum*	*Capsicum annuum*	Chili pepper	South America, northern Peru	Condiment and medicinal
**Solanales**	Solanaceae	*Nicotiana* *	*Nicotiana sylvestris*	Tobacco	Probably Mexico, Central America	Narcotic and hallucinogenic
**Solanales**	Solanaceae	*Solanum* *	*Solanum lycopersicum*	Tomato	Western South America	Foodstuff
**Malvales**	Malvaceae	*Gossypium* *	*Gossypium barbadense*	Cotton	Northwestern Peru and southwestern Ecuador [54]	Fiber and oil

*Putative taxonomic assignment of plant sequences in the present dataset.

^a^ Data obtained from [55].

### Starchy tubers, legumes, pseudograins, fruits, and a hallucinogenic plant were likely part of the Huecoid and Saladoid vegetal diet and culture

**Fig 1:**
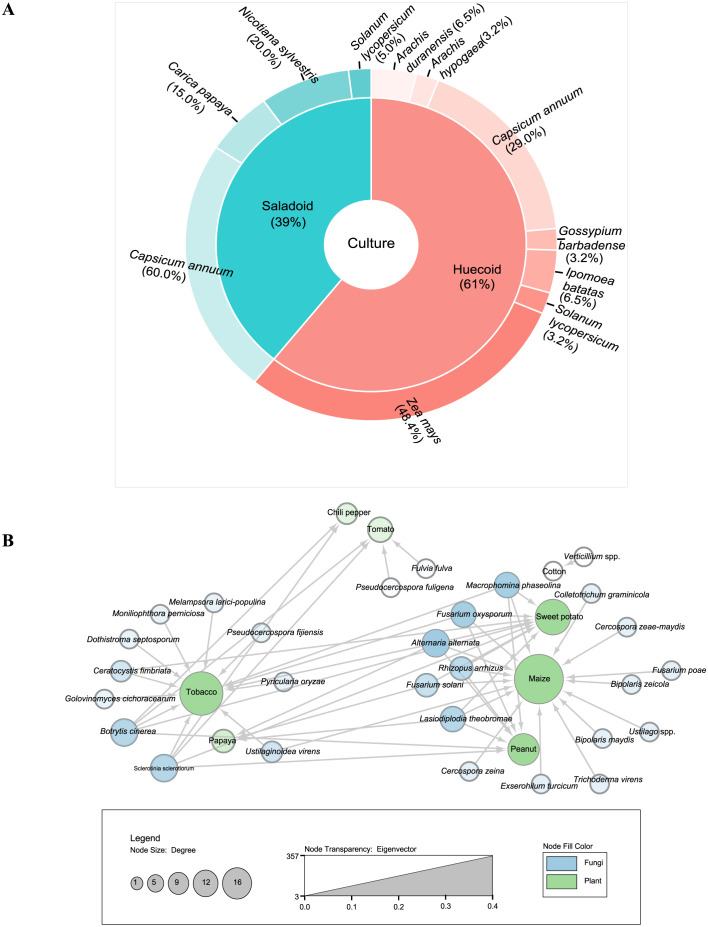
Piedonut diagram for plant distribution and directed network analysis of pathogen-host interactions of the pre-Columbian Huecoid and Saladoid cultures. Panel **(A)** Inner pie chart represents the percentage of plants identified by culture, whereas the outer donut shows the distribution of the plants. Panel **(B)** Pathogen-host interaction network constructed using the rglobi (global biotic interactions) database. Relationships between fungal pathogens and plant hosts are represented as directed edges from source (fungi) to target (plants). Each node represents either plant (green) or fungal (blue) taxa. Plant node size represents indegree and plant node transparency depicts the Eigenvector centrality.

Plant sequencing reads in this study revealed a variety of food plants in the Huecoid and Saladoid coprolites. We found a high abundance of maize (*Zea ma*y*s*; relative abundance = 48.4%) followed by chili pepper (*Capsicum annuum*; 29.0%), sweet potato (*Ipomoea batatas*; 6.5%), wild peanut (*Arachis duranensis*; 6.5%), domesticated peanut (*Arachis hypogaea*; 3.2%), cotton (*Gossypium barbadense*; 3.2%) and tomato (*Solanum lycopersicum*; 3.2%) in the Huecoid coprolite sample. Notably, we observed less plant DNA sequences Saladoid coprolite sample compared to the Huecoid coprolite sample. Specifically, plant sequence reads identified in the Saladoid coprolite sample included chili pepper (*Capsicum annuum*; 63.2%), tobacco (*Nicotiana sylvestris*; 21.1%), papaya (*Carica papa*y*a*; 15.8%), and tomato (*Solanum lycopersicum*, 5.0%). Sequencing reads of chili peppers and tomato were shared between the Huecoid and Saladoid coprolite samples, while seven plant taxa were only identified in one sample (Fig 1A).

Using network analysis, we examined the relationship between fungal pathogens and plant hosts in the coprolites of the Huecoid and Saladoid cultures, considering previous archaeological findings. Due to the limited taxa in the Saladoid coprolite sample, we merged the two ethnic groups and created clusters in the network model. The maize node had the highest degree of connectivity (degree = 16) and eigenvector centrality (eigenvalue = 0.39), indicating that maize could potentially host many fungi and plays a critical role in connecting other nodes. The tobacco node had the second highest degree of connectivity (degree = 13), followed by sweet potato (degree = 9), and peanuts (degree = 7). However, sweet potato had a higher eigenvector centrality (eigenvalue = 0.35) than tobacco (eigenvalue = 0.34), suggesting that sweet potato is more influential in the network. Plants with the lowest degree of connectivity and eigenvector centrality were Papaya (degree = 3, eigenvalue = 0.12), tomato (degree = 4, eigenvalue = 0.08), chili pepper (degree = 2, eigenvalue = 0.08), and cotton (degree = 1, eigenvalue = 0) (Fig 1B).

### Phytocultural practices of ancient cultures may be different from those of present-day cultures

**Fig 2:**
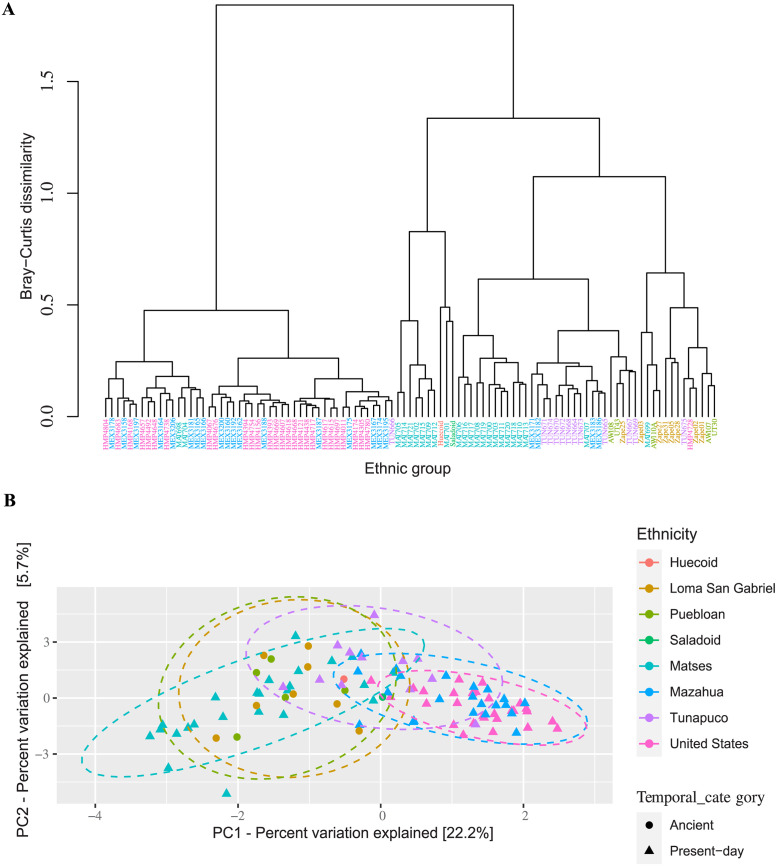
Composition and structure of plants differentiate the ethnic groups according to dietary lifestyles. Panel **(A)** Bray-Curtis distance dendrogram constructed on plant family abundance showing hierarchical clustering/relationships between similar samples. Panel **(B)** Principal component analysis of Aitchison distances showing that plant families-diversity segregated pre-Columbian ethnic groups and hunter-gatherers from present-day large-scale farmers and industrialized individuals, whereas the agriculturalists showed a transitional state. Each color code represents the ethnicity of each group, whereas the circle and triangles symbols represent plant communities of each sample in ancient and present-day ethnic groups, respectively. Adonis test was performed on Aitchison distances.

For comparative purposes, we analyzed publicly available coprolite sequence data from Rio Zape Cave (Mexico), Boomerang Shelter (United States), and Arid West Cave (United States) as well as present-day feces from the Matses hunter-gatherers (Peru), Tunapuco (Peru) and Mazahuas (Mexico) agricultural communities, and industrial populations (from the United States). We quantified Bray-Curtis dissimilarity and Aitchison distances to investigate differences in the plant beta diversity across the samples. Hierarchical cluster analysis based on Bray-Curtis dissimilarity, and the plant relative abundance of each sample reflected two main clusters; combining present-day feces from Mazahua and United States (Cluster 1); and coprolites from Ancestral Puebloans, Loma San Gabriel, Huecoid and Saladoid as well as present-day feces from Matses and Tunapuco (Cluster 2) (Fig 2A). PCoA based on Aitchison distances and relative abundance of plant families showed significant segregation (PERMANOVA, R2 = 0.27 and p-value = 0.001) in the plants across samples from the Amerindian groups (Fig 2B), suggesting differences in community structure. The PCoA axis 1 (PC1) explained 22.2% of the variance, which correlated with a gradient in human lifestyles. For instance, the pre-Columbian cultures and present-day hunter-gatherers were located on the left, the Tunapuco agriculturalists in the middle and the Mazahua farmers and United States individuals on the right (Fig 2B). Thus, despite differences in geography, coprolites from Arid West, Boomerang Shelter, Loma San Gabriel, Huecoid, and Saladoid were more similar to each other, and to present-day feces from Matses hunter-gatherers than to present-day feces from Mazahua and the United States (Fig 2B). However, the Tunapuco feces showed a transitional state between the coprolites and present-day feces from hunter-gatherers, and the present-day feces from the Mazahua farmers and individuals from the United States. The findings indicate a shift in behaviors leading to modifications in dietary patterns (Fig 2B).

## Discussion

Through the integration of molecular data, pathogen-host interaction modeling, and published literature, we present the first attempt to reconstruct the plant-based diets of pre-Columbian cultures in Puerto Rico using preserved ancient plant and phytopathogen DNA sequences. We analyzed plant sequence reads obtained from coprolites of the Huecoid and Saladoid cultures and confirmed the identity of plant DNA using phytopathogenic fungi DNA that may have impacted their horticultural ecosystem. We also compared our results with previously published coprolite sequence data from Mexico and the United States, as well as present-day fecal samples from Mexico, Peru, and the United States, to investigate the phyto-cultural diversity between ancient and present-day populations in various social environments. Our study provides insights into the lifestyle and dietary habits of the Huecoid and Saladoid cultures, which have contributed to the cultural identity of present-day Caribbeans, by identifying the plants used for consumption by these pre-Columbian groups. Furthermore, our findings suggest that the incorporation of native plant sequences into DNA databases is crucial to use DNA-based approaches for reconstructing dietary habits. Although historical records of the plants used in the Huecoid and Saladoid cultures are scarce, it is possible that closely related plant phyla and families served as food for these ethnic groups and could be extinct, replaced, and forgotten due to the introduction of other crops resulting from the colonization of the Americas.

### Overcoming challenges with contamination

Sample contamination with modern exogenous DNA is a major challenge in the analysis of ancient DNA from coprolites [56, 57]. We used SourceTracker to test for contamination and verify the authenticity of coprolite DNA, and found that unknown sources contributed the highest number of eukaryotic sequences (S1 Fig). This may suggest either that no soil contamination affected the results, or that other sources not included in the mSourceTracker analysis may have contributed to the results in the present study. Moreover, the high proportion of unknown sources contributing taxa is consistent with previous studies on coprolites and mummies [30, 38]. Interestingly, we also found that published metagenomes of coprolites were the main known sources contributing to the Huecoid coprolite samples, suggesting that the Huecoid eukaryote sequences are endogenous to the coprolite sample. In contrast, soil was the primarily source contributing to the Saladoid coprolite, followed by coprolite samples. This may suggest that the coprolite samples were possibly contaminated with soil as a result of being exposed to the environment, or vigorously washing edible plants was potentially not practiced by pre-Columbian cultures. Another plausible explanation may rely on soil as an important microbial seeding source (possible geophagy) [58]. Geophagy (ingestion of soil intentionally or unintentionally), has been used by humans to protect them from dietary chemicals and pathogens [59]. It has been observed in many different cultures around the world [59], and archaeological evidence suggests that geophagy dates back to *Homo habilis* [60, 61].

### Evidence of various food items

Earlier research has revealed a wide range of plants that were processed using lithic artifacts such as *burén*, stones, and shells, as evidenced by the presence of preserved starch grains [19]. This suggests a sophisticated food system, which is in contrast to historical accounts that emphasize the indigenous cultures’ dependance on manioc [62–65]. Consistent with this early archaeobotanical study, we identified DNA sequences from a diversity of plants, including a starchy tuber (sweet potato), legume (peanut), solanaceous fruit (chili peppers and tomato), caricaceous fruit (papaya), pseudograin (maize), and other crops (tobacco and cotton). Such plants identified in the Huecoid and Saladoid coprolites analyzed suggest a variety of dietary, medicinal, and hallucinogenic plants as part of these pre-Columbian cultures diet and culture. Although very useful and insightful, the results and conclusions in the present study are limited by the fact that there are relatively few available plant genomes sequenced and available in the current databases. As the number of genome sequences increases, sequences obtained from coprolites will probably be more defined.

### Tobacco

Tobacco originates in the New World, particularly in Central and South America [66]. It has been reported that indigenous cultures from the 16^th^ century would consume tobacco for hallucinogenic, as well as medicinal purposes as it would alleviate pain and induce sleep [66]. It has also been hypothesized that tobacco was consumed in social activities [66]. Consumption of tobacco comes from chroniclers dealing with pre-Columbian cultures, such as the Aztecs [66], the Maya [67] and indigenous people from La Hispaniola [13, 14]. Traces of nicotine were detected in a Late Mayan period flask (Campeche, Mexico) dating around 700 AD by using chromatography and mass spectrometry, indicating the ancient use of tobacco in the Mayan culture [67]. The Mayan flask may have been used for tobacco enema preparations, likely for rituals and medicinal purposes [68]. However, limited records exist on tobacco consumption by other pre-Columbian cultures. Nevertheless, information from specific pre-Columbian cultures may provide insights into tobacco consumption in the Huecoid and Saladoid cultures. As such, there are several plausible practices that may explain the presence of tobacco sequences in the Huecoid and Saladoid coprolite samples. First, tobacco may be chewed, and although we could not find any references of this practice in pre-Columbian records, there is a likelihood that tobacco could have been consumed in this manner. An alternative explanation for the presence of tobacco sequences in the coprolite samples include the use of wood or ceramic inhalers, where pulverized tobacco (and other herbs, or mixtures) can be placed. The inhaler is then inserted in the nostrils of the recipient and a second person would blow into the inhaler to force the powder deep into the nostrils. Tobacco could also have been used as an additive for food and drink [67].

### Sweet potato and legumes

Sweet potato and legumes (including common beans) played an important function in the agricultural economies of ancient Puerto Rico [18]. We identified DNA sequences likely corresponding to sweet potato and legumes in the Huecoid coprolite sample. Pre-Columbian cultures relied on specific food items such as sweet potato, which would be consumed with fruits, vegetables, meat, fish [5, 17], mollusks, and crustaceans. In addition to sweet potato sequences, we observed Fabaceae sequences (putatively assigned as peanut), suggesting that it may have been a component of the Huecoid diet. Indeed, peanuts are native to the New World, originating in South America, and new species continue to be discovered [69]. Sweet potato and legumes have also been found on lithic and shell artifacts, and dental calculus from Puerto Rico and other regions of the Caribbean [18, 19, 70, 71], suggesting the consumption of these plant items by pre-Columbian cultures. Legumes persisted in the ancient Caribbean diet of pre-Columbian cultures from the early ceramic age to the early colonial period, whereas the sweet potato was a key starchy crop during the pre- and post-Columbian eras [18]. The low abundance of sweet potato sequences in the Huecoid coprolite sample may be a result of food traces from a previous meal that were obscured by the abundant foods of the latest meal [21], or the sporadic consumption of this item.

### Maize

Maize (*Zea Ma*y*s*), a plant domesticated in Mesoamerica [72, 73], was introduced from the circum-Caribbean region (Central America and the northern countries of South America) to Puerto Rico probably during the archaic age approximately 5,000 B.P [74]. Early European chroniclers indicate that indigenous cultures cultivated maize twice a year and consumed it tender, as well as raw, and roasted. They also included maize in certain stews, and would also consume it ground and with water [13]. Maize could be ground or pounded and further baked, grilled, or toasted by these pre-Columbian cultures to possibly prepare bread [15, 75]. While maize has been previously considered a restricted crop [17, 76], evidence of human isotope and pre-Columbian dental calculus from Puerto Rico and the Caribbean suggest that maize was frequently consumed [15, 77]. Such findings were further extended by Pagan and Mickleburgh, who suggested that maize was the most ubiquitous edible crop of the insular Caribbean [18]. Overall, these results suggest that maize had an important role in pre-Columbian dietary habits. We detected a high abundance of maize in the Huecoid coprolite sample, suggesting that maize was an important crop in this culture, likely consumed daily, which is consistent with previous paleomicrobiological findings [32]. In addition, the analysis of starch residues in lithic tools from two Huecoid settlements in Puerto Rico demonstrated that the Huecoid culture maintained and used this plant [19]. The detection of certain plants, like maize, exclusively in the Huecoid culture may further support differences in cultural backgrounds observed in archeological records, as well as microbiome, virome and mycobiome analyses. However, perhaps the Saladoid culture consumed maize sporadically and thus, sequences were not detected in these particular coprolite samples. Moreover, the presence of *Ustilago* spp. sequence reads, a fungal genus known to infect maize, in the coprolites not only provided further evidence of maize consumption, but possibly point to the consumption of huitlacoche (musuro or sara musuru in quechua) [78], a common fungal phytopathogen that is prized as a delicacy, even by today’s cultures.

### Chili peppers

Chili peppers have been used for food, medicinal and religious purposes throughout the Americas [55]. Paleo-biolinguistics along with genetic and archaeobotanical evidence have shown that domesticated chili pepper originated in central-east Mexico approximately 6,500 years ago [79]. Chili peppers are not frequently found in the archaeological record likely due to poor starch or capsain resiliency over time [80]. However, starches of chili pepper have been detected in food-processing tools of the early southern Caribbean and the late pre-Columbian period of the northern Caribbean [18, 70], and were likely consumed as a condiment, stimulant and medicine in the pre-Columbian era [13, 14]. We identified sequencing reads matching chili peppers in both the Huecoid and Saladoid coprolites. Notably, it has been shown that chili peppers and maize occurred together in food-processing tools, suggesting a preferred food-complex [80]. Consistent with these observations, we found a high abundance of maize and chili pepper DNA sequences in the Huecoid coprolite sample.

### Unexpected findings and absence of expected sequences

#### Tomato

Tomato is currently divided into *S*. *lycopersicum* var. *lycopersicum* and *S*. *lycopersicum* var. *cerasiforme*, and *S*. *pimpinellifolium* is considered the most closely related wild species of tomato [81]. The roles of some of these species in the domestication of tomato remain unclear and a matter of further investigation as some consider that *S*. *l*. *cerasiforme* is an ancestor of tomato, while others consider that *S*. *l*. *cerasiforme* is a combination of *S*. *pimpinellifolium* and *S*. *l*. *lycopersicum* [81]. While it remains unclear if the domestication of tomato occurred in the Andean region or Mesoamerica, one study suggested that pre-domestication occurred in the Andean region, and domestication occurred in Mesoamerica [81]. The presence of *Solanum* spp. (tomato) DNA sequences was somehow unexpected; however, this food item may have been brought along with other food crops from South America.

#### Cotton

The presence of *Gossypium* spp. (cotton) sequences was unexpected since it is a non-edible crop used for textile throughout the ages. However, a possible explanation might be the use of the seeds as either additives or as source of oils to be used in some manner; although cotton oils are known for their bitter flavors. Nonetheless, this finding opens up more questions than it answers. Current cotton oils are not likely to have DNA present, but that is because of the highly processed nature of these oils. These ethnic groups may have found the ground seeds were a food additive to their diet in some manner. Other possible explanation is that indigenous women ingested cotton fibers during the weaving process by using the saliva to prepare the raw yarn. In fact, cellulose fibers of cotton have been found in dental calculus from the Late Woodland period (900–1100 AD) of the Danbury site (Ohio), suggesting the processing of cotton fibers for textiles and likely fishing nets using the mouth [82]. Cotton fibers have been found to contain ample concentrations of DNA [83]. Additionally, cotton processing and textile crafting was (and still is in many cultures) a female-only activity; thus, a likely scenario is that these coprolites were deposited by female members of the ethnic groups.

#### Cassava

Many archaeological narratives of the Caribbean suggest that the subsistence strategies of the Huecoid and Saladoid cultures were primarily based on cassava/yucca/manioc (*Manihot esculenta*) [62–64]. Cassava can be “sweet” or “bitter”, with the later retaining its toxic liquid if not extracted [84, 85]. While “sweet” cassava has been selected to be non-toxic and can be consumed either boiled or roasted, most cultivated types are “bitter” and retain the toxicity and as such require processing to extract the toxic liquid [84, 85]. The chroniclers widely described the “bitter” cassava in the Antilles and reported it consumption by indigenous people after a long preparation process that includes grating and squeezing the cassava pulp with a *sebucán* to extract the toxic liquid, followed by a drying stage to produce flat tortas [14]. The chroniclers also reported that “sweet” cassava was not observed in the Antilles but on the mainland. The absence of cassava DNA sequences in the coprolite samples may suggest the extensive pretreatment of *Manihot* spp. to remove toxins contained in the liquid. Indeed, certain methods of food preparation, are known to degrade dietary DNA [34, 86], which could then be further degraded by enzymes and microbes during digestion [87], as well as by taphonomic processes. Alternatively, the absence of some plants DNA could also explain seasonal variation [26], or sporadic consumption of certain food items, such as cassava.

The importance of cassava has been debated over the years. One study including the analysis of Huecoid lithic tools from La Hueca, Vieques, showed the recovery of ancient cassava starches from a single tool [19]. In contrast, sweet potato, and other plants (including maize), were identified in several lithic tools, suggesting that cassava was only part of a diverse spectrum of plants contributing to the diet [19]. Although archaeological narratives suggest that cassava was introduced to Puerto Rico by the Saladoid, cassava starch grains were undetected in twenty-four tools corresponding to this culture [88, 89]. Similarly, cassava starches were unidentified in “burenes” (tools often associated with the cooking of cassava) from the Saladoid culture, where sweet potato and other plant remains (maize and beans and others) have been frequently found.

### Spatiotemporal dietary variations

Pre-Columbian cultures and present-day hunter-gatherers showed contrasting diets compared to present-day agriculturalists and urban-industrial individuals. Plant communities among the ethnic groups were significantly segregated based on ethnicity and lifestyles, as shown by hierarchical clustering and PCoA. Since the pre-Columbian cultures inhabited regions similar to those of their present-day counterparts, it is unlikely that changes in dietary habits are due to geography. Clustering of plant communities based on lifestyles (i.e., hunter-gatherer, agriculturalist and urban-industrial) suggested changes in dietary habits in response to transitions in human lifestyles. In fact, the diet of past populations is known to differ greatly from that of modern populations depending on the environment, socioeconomic status, and available resources [90]. During the Neolithic Era, human dietary lifestyles transitioned from game meat and gathering of unprocessed fruits from the environment (i.e., hunter-gatherers) [91], into one based on agriculture and animal domestication (farming). However, a westernized diet was adopted with the industrial revolution, characterized by being high in fats and including simple carbohydrates [92, 93].

Ancestral Puebloans (the Anasazi) were a prehistoric culture from the Colorado Plateau, which include the States of Colorado, New Mexico, Arizona, and Utah. Macro-remains analysis of coprolites from the Arid West (Arizona) and the Boomerang Shelter (Utah) have shown that maize-derived foods (including huitlacoche) [23, 94], and prickly pear fruit (*Opuntia*) are abundant components of the Ancestral Pueblo diet [23]. In contrast, coprolites from the Loma San Gabriel culture, a prehistoric population from Rio Zape Valley in Durango, Mexico, showed that they subsisted mainly on Agave and maize [21, 95]. Other plants that supplemented their diet included squash (*Cucurbita* spp.) and beans (*Phaseolus* spp.). In agreement with previous studies, our study showed that the Huecoid and Saladoid plant diet consisted of starchy tubers, maize, and legumes, supplemented with fruits. It is well known that food sources can vary due to differing geographical and cultural characteristics. However, the Huecoid and Saladoid shared food sources with the Ancestral Pueblo culture and the Loma San Gabriel culture, which may explain the clustering patterns. Nonetheless, this study is biased towards domesticated taxa, whose sequences are available in databases. Thus, food sources identified in geographically disparate pre-Columbian cultures could be showing the importance of domesticated crops in human diets after being adopted by these cultures. Notably, the presence of certain plant sequences may also correspond to what was consumed a short period prior to fecal deposition. Despite the fact that coprolites provide relevant information about diet, food plant DNA in each coprolite likely reflects a few previous meals prior to defecation.

Interestingly, the present-day Matses also seems to be more similar to the Ancestral Puebloans, Loma San Gabriel, Huecoid and Saladoid pre-Columbian cultures, despite differences in culture and temporal scales. The Matses hunter-gatherers have a diet mainly composed of gathered tubers (*Manihot* spp.) and plantains (*Musa* spp.) [41]. On the other hand, the Tunapuco dietary lifestyle suggests a state of transition from hunter-gathering to agriculture. Potatoes (*Solanum tuberosum* spp.), oca (*Oxalis tuberosa*), and mashua (*Tropaeolum tuberosum*) are part of every meal of these extant agriculturalists from the Peruvian highlands [41]. In contrast, the Mazahua farmers from Mexico had a greater resemblance to the United States individuals, likely due to a higher agricultural production in the Mazahua community compared to the Tunapuco agriculturalists. The present-day Mazahua farmers base their diet on maize, secondarily on wheat and edible mushrooms [23, 96]. Individuals from the United States exhibit the typical western diet composed of processed foods and dairy products [42]. Although rare or limited, the Tunapuco consume dairy and processed foods [41]. In addition, rice and bread are the main food supplementing the Tunapuco diet, while wheat contributes the majority of the Mazahua calories after maize. Similar dietary components among the Mazahua and United States feces, and to a lesser extent the Tunapuco, may have resulted in the clustering patterns observed for the Mazahua and United States and the transitional state of the Tunapuco. These results are partially supported by a recent study showing the segregation of pre-Columbian and present-day populations based on their gut mycobiome [36]. Studies have shown that dietary lifestyles strongly shape the composition of the gut microbiome of traditional populations, which differs from that of industrialized populations [41, 97–104].

The detection of plant DNA in coprolites may be biased towards foods that are consumed raw or lightly cooked, as cooking and food preparation can result in the liberation of DNA form cells. Additionally, plant materials that have been metabolized during digestion may be difficult to identify [105, 106]. Furthermore, the degradation of DNA by nucleases during digestion can also affect the results. Maize was commonly identified in the Huecoid coprolites likely due to non-digestible fibers that are resistant to digestion [107]. Identifying certain plant sequences can be challenging due to limitations in current DNA databases that primarily include plants of commercial and economic importance. Taphonomic processes that damage ancient DNA can also aggravate and restrict the matching of ancient DNA sequences to those available in existing databases. It is important to note that a match or close hit between sequences does not necessarily imply similarities across the species being compared, but rather that the ancient DNA sequence could represent a taxon that is not represented in the database [26].

## Conclusions

We conducted DNA sequence analyses of plant sequencing reads from coprolites, which revealed the presence of a variety of plants in the Huecoid and Saladoid cultures of the Caribbean, as well as in ancient and present-day America. Our study supports archaeological records that suggest that these cultures consumed maize (*Zea mays*), sweet potato (*Ipomoea batatas*), chili pepper (*Capsicum annuum*), papaya (*Carica papaya*), peanut (*Arachis* spp.), tobacco (*Nicotiana sylvestris*), tomato (*Solanum lycopersicum*), and surprisingly, cotton (*Gossypium barbadense*). However, it is important to note that coprolites reflect a limited range of potential plants ingested, and the detection of plant sequences in a single culture may suggest preferences for certain plants, occasional consumption, degradation due to specific food preparation practices, or taphonomic processes.

Our analyses are limited by the current DNA sequence databases, which are focused mostly on commercially important crops. Expansion of these databases to include plants that are not necessarily of economic importance might allow us to better understand ancient dietary habits, as well as those of extant remote populations. By examining plant and fungi sequences, our data aids in the reconstruction of the dietary habits and lifestyles of the Huecoid and Saladoid cultures, and opens the opportunity to further understand the diets and lifestyles of other pre-Columbian groups in America.

## Supporting information

S1 Table Detailed description of the archaeological samples analyzed in this study. (DOCX)

S1 Fig Source proportion estimates for the Huecoid and Saladoid coprolite samples (sink) using reference datasets of environmental samples (source). Meta-SourceTracker showed the proportion of Eukaryote domain sequencing data that each environmental source sample contributed to the Huecoid and Saladoid coprolite sink samples. Overall, mSourceTracker showed that unknown sources contributed the highest proportions of Eukaryote reads in the Huecoid (0.41%) and Saladoid (0.68%) coprolites. Besides unknown sources, mSourceTracker estimated that a high proportion of the eukaryote reads of the Huecoid coprolite sink sample came from well-preserved coprolite source samples (0.33%). Conversely, a high proportion of eukaryotes exhibited soil (0.24%) and coprolite (0.07%) origin in the Saladoid coprolite sink sample.
(PDF)
