# Supplementary material for: Edible flora in pre-Columbian Caribbean coprolites: Expected and unexpected data
Source: PLoS One. 2023 Oct 11;18(10):e0292077. doi: 10.1371/journal.pone.0292077 (PMC10566737; doi:10.1371/journal.pone.0292077)
Supplement: S1 Table — (DOCX) [file pone.0292077.s001.docx]

| Sample ID | Archaeological site | Geographical region | Radiocarbon date | Reference |
| --- | --- | --- | --- | --- |
| Huecoid | La Hueca, Sorcé | Vieques, Puerto Rico | 470 A.D. | This study, (34,35) |
| Huecoid | La Hueca, Sorcé | Vieques, Puerto Rico | Circa 385 A.D. | This study, (34,35) |
| Huecoid | La Hueca, Sorcé | Vieques, Puerto Rico | Circa 450 A.D. | This study, (34,35) |
| Huecoid | La Hueca, Sorcé | Vieques, Puerto Rico | Circa 245 A.D. | This study, (34,35) |
| Huecoid | La Hueca, Sorcé | Vieques, Puerto Rico | 215-220 A.D. | This study, (34,35) |
| Huecoid | La Hueca, Sorcé | Vieques, Puerto Rico | 470-600 A.D. | This study, (34,35) |
| Saladoid | La Hueca, Sorcé | Vieques, Puerto Rico | 270-385 A.D. | This study, (34,35) |
| Saladoid | La Hueca, Sorcé | Vieques, Puerto Rico | 230-385 A.D. | This study, (34,35) |
| Saladoid | La Hueca, Sorcé | Vieques, Puerto Rico | 230-385 A.D. | This study, (34,35) |
| Saladoid | La Hueca, Sorcé | Vieques, Puerto Rico | 335-395 A.D. | This study, (34,35) |
| UT30.3 | Boomerang Shelter | Utah, USA | 60 A.D. | (23) |
| UT43.2 | Boomerang Shelter | Utah, USA | 10 A.D. | (23) |
| AW107 | Arid West Cave | Arizona, USA | 595 A.D. | (23) |
| AW108 | Arizona Cave | Arizona, USA | 635 A.D. | (23) |
| AW110A | Arid West Cave | Arizona, USA | 620 A.D. | (23) |
| Zape1 | La Cueva de los Muertos Chiquitos, Rio Zape | Durango, Mexico | 920 A.D. | (23) |
| Zape2 | La Cueva de los Muertos Chiquitos, Rio Zape | Durango, Mexico | 850 A.D. | (23) |
| Zape3 | La Cueva de los Muertos Chiquitos, Rio Zape | Durango, Mexico | 725 AD | (23) |
| Zape 5 | La Cueva de los Muertos Chiquitos, Rio Zape | Durango, Mexico | 1300 BP | (39) |
| Zape 25 | La Cueva de los Muertos Chiquitos, Rio Zape | Durango, Mexico | 1300 BP | (40) |
| Zape 27 | La Cueva de los Muertos Chiquitos, Rio Zape | Durango, Mexico | 1300 BP | (40) |
| Zape 28 | La Cueva de los Muertos Chiquitos, Rio Zape | Durango, Mexico | 1300 BP | (39) |
| Zape 31 | La Cueva de los Muertos Chiquitos, Rio Zape | Durango, Mexico | 1300 BP | (40) |
