# Supplementary figures and images for: Edible flora in pre-Columbian Caribbean coprolites: Expected and unexpected data
Source: PLoS One. 2023 Oct 11;18(10):e0292077. doi: 10.1371/journal.pone.0292077 (PMC10566737; doi:10.1371/journal.pone.0292077)

Mixing Proportions (as Fraction)

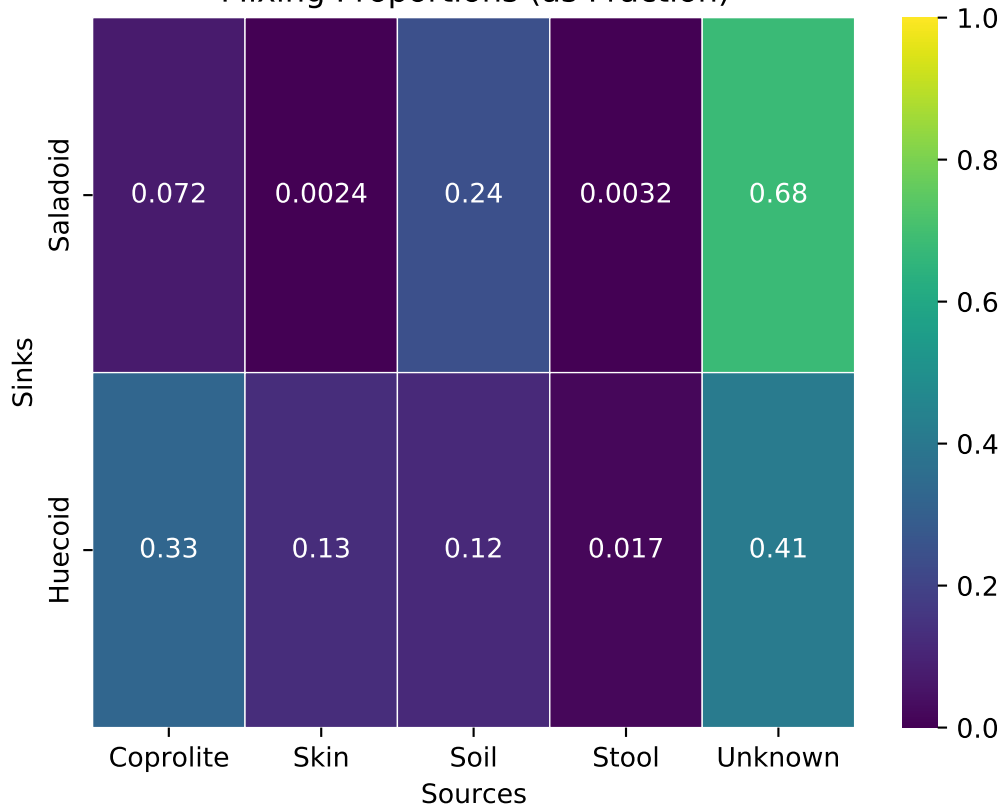

Supplement: S1 Fig — Meta-SourceTracker showed the proportion of Eukaryote domain sequencing data that each environmental source sample contributed to the Huecoid and Saladoid coprolite sink samples. Overall, mSourceTracker showed that unknown sources contributed the highest proportions of Eukaryote reads in the Huecoid (0.41%) and Saladoid (0.68%) coprolites. Besides unknown sources, mSourceTracker estimated that a high proportion of the eukaryote reads of the Huecoid coprolite sink sample came from well-preserved coprolite source samples (0.33%). Conversely, a high proportion of eukaryotes exhibited soil (0.24%) and coprolite (0.07%) origin in the Saladoid coprolite sink sample. (PDF) [file pone.0292077.s002.pdf]
